# Comparison of endoscopic and microscopic microvascular decompression for treating primary trigeminal neuralgia

**DOI:** 10.3389/fneur.2025.1583192

**Published:** 2025-07-02

**Authors:** Lei Li, Dongqi Shao, Xialin Zheng, Yuanbo Pan, Tao Sun, Huadong Tang, Hongjie Zhai, Xiaohui Dong, Jing Sun, Mengtian Fang, Feiyun Lou, Zhiquan Jiang

**Affiliations:** ^1^Anhui Medical University, Hefei, China; ^2^Department of Neurosurgery, The First Affiliated Hospital of Bengbu Medical University, Bengbu, China; ^3^Department of Neurosurgery, The Second Affiliated Hospital Zhejiang University School of Medicine, Hangzhou, China

**Keywords:** endoscopy, microscopy, neuroelectrophysiological monitoring, microvascular decompression, primary trigeminal neuralgia

## Abstract

**Objective:**

To compare the intraoperative outcome and postoperative efficacy of endoscopic and microscopic microvascular decompression (MVD) under neuroelectrophysiological monitoring in the treatment of primary trigeminal neuralgia (PTN).

**Methods:**

This retrospective study included 131 patients with PTN who were admitted to our hospital between January 2019 and October 2022. The patients were categorized according to the treatment received into the endoscopic group (*n* = 53) (endoscopic MVD) and the microscopic group (*n* = 78) (microscopic MVD). All patients were treated under neuroelectrophysiological monitoring. The surgical time, identification of offending vessels, full exposure of trigeminal nerve (TN) structure, the rate of one-time decompression, postoperative efficacy, complications, postoperative hospital stay and recurrence rate were compared between both groups.

**Results:**

There were differences in incision length and bone flap diameter between the two groups (*p* < 0.05). Endoscopy was more minimally invasive compared to microscopy; however, there was no significant difference in total surgical time and blood loss between the two groups (*p* > 0.05). In the group with no neurovascular compression identified during preoperative assessment, the surgical duration was significantly shorter with endoscopy compared to microscopy (*p* < 0.05), which indicated that endoscopic treatment has a time advantage in this condition. The consistency rate of preoperative magnetic resonance angiography (MRA) result and intraoperative offending vessels differed between the two groups (*p* < 0.05), indicating that endoscopy may be more accurate than microscopy in detecting offending vessels. Compared to microscopes, endoscopes provide a higher rate of full exposure of the TN (*p* < 0.05). This difference is primarily observed in cases where there is obstruction by the petrosal protuberance tubercle (PPT) or petrosal vein (PV). Additionally, endoscopes offer a higher rate of one-time decompression which means that the need to adjust the Teflon pledget is less frequent (*p* < 0.05), thereby reducing the number of nerve disturbances. There was no significant difference in postoperative remission rate and Barrow Neurological Institute Pain Intensity Score (BNI score) between the groups (*p* > 0.05). Compared to the microscopy group, the recurrence rate at 2 years and the last follow-up after endoscopic surgery was lower; however, the difference was not significant (*p* > 0.05). There was no significant difference in the incidence of postoperative complications, mortality rate, and length of hospital stay between the groups (*p* > 0.05).

**Conclusion:**

Endoscopic and microscopic MVD are effective for PTN and have comparable outcomes. Endoscopy enhances visualization and identification of offending vessels; moreover, compared with microscopy, endoscopy is more minimally invasive and suitable for detecting and fully separating potentially hidden offending vessels; however, its technical complexity necessitates individualized treatment based on patient needs and institutional expertise.

## Introduction

Primary trigeminal neuralgia (PTN) is a neurological disorder characterized by severe pain in the distribution of one or more branches of the trigeminal nerve (TN). The incidence rate of PTN is 2.1–27 cases/100,000 people and increases with age (1–5). PTN seriously affects the patient’s quality of life as it can be associated with severe pain, even leading to depression and suicidal tendencies (1, 4, 5). Microvascular decompression (MVD) is the most effective and widely recognized first-line surgical treatment for PTN (2, 4, 6, 7). Eby et al. (8) pioneered the use of endoscopic MVD (E-MVD) to treat PTN and achieved satisfactory results. Since then, endoscopic technology has been increasingly used in MVD surgery by neurosurgeons and many articles are devoted to the comparative study of endoscopic and microscopic MVD (M-MVD) due to its unique and adequate field of view. However, most of them are limited to discussing the improved field of view offered by endoscopy, and there is a lack of comparative data to substantiate its superiority in this regard (6–8). This study aims to compare and explore the differences in intraoperative situation and therapeutic efficacy between E-MVD and M-MVD for the treatment of PTN, as well as their respective advantages and limitations, in order to provide more references for the surgical treatment of PTN.

## Materials and methods

### General information

This retrospective study included 131 patients with PTN who received MVD in our hospital between January 2019 and October 2022. All surgeries were performed by the same surgeon. Based on the preoperative evaluation of surgical safety and effectiveness, when there is no significant difference between endoscopic and microscopic procedures, the selection of the surgical approach considers patient preference. Overall, 53 patients underwent fully endoscopic surgery without the use of a microscope at any stage, while 78 cases underwent microscopic surgery. All patients were treated using the suboccipital retrosigmoid sinus approach and underwent neuroelectrophysiological monitoring throughout the surgery. Routine monitoring using trigeminal free electromyography (FreeEMG) and brainstem trigeminal evoked potential (BTEP) was also performed. The Barrow Neurological Institute Pain Intensity Score (BNI score) was used to grade the severity of facial pain. Long-term postoperative efficacy was assessed through outpatient follow-ups and telephone interviews, with an average follow-up duration of 40.44 ± 13.35 months (range, 24–68 months).

### Inclusion and exclusion criteria

The study’s inclusion criteria were as follows: (1) Patients who underwent enhanced MRI before surgery to exclude secondary trigeminal neuralgia, and magnetic resonance angiography (We use the Three-dimensional Time-of-Flight Fat Saturation Spoiled Gradient Recalled Echo (3D TOF-FS-SPGR) sequence for evaluation. Hereinafter, it is referred to as ‘MRA’) confirmed the presence or absence of offending vessels, diagnosed with PTN; (2) Patients with typical symptoms of trigeminal neuralgia; (3) Patients who underwent surgery performed by experienced surgeons with more than 10 years of endoscopic and microscopic surgery; (4) Patients with unilateral onset who had not previously undergone other surgical or radiation treatments preoperatively but had received pharmacological treatment were included.

The study’s exclusion criteria were as follows: (1) Patients who underwent preoperative radiofrequency therapy, percutaneous balloon compression surgery, or MVD were excluded from this study; (2) Patients with other systemic malignancies; (3) Patients with incomplete preoperative and postoperative data, loss to follow-up, or death/severe disability due to other reasons; (4) The endoscope-assisted microscopic surgery was excluded during the study period.

### Methods

#### Surgical procedure steps

The scalp was prepared, and general anesthesia was administered. The patient was then placed in the lateral oblique position (park bench position) with the healthy side facing downwards. The head was appropriately positioned on the operating table using the Mayfield head holder, and was adjusted to an appropriate angle to ensure that the mastoid process on the affected side was at the highest position while ensuring unobstructed blood flow in the neck. Connect neuroelectrophysiological electrodes to monitor trigeminal FreeEMG and BTEP. A longitudinal skin incision (3–5 cm in length) was made within the hairline of the posterior digastric groove, and the muscles were separated. A milling cutter was then used to mill a bone flap with a diameter of 2–3 cm at the junction of the transverse and sigmoid sinuses. If the mastoid air chamber was open, it was rinsed repeatedly using hydrogen peroxide, dilute iodine, and physiological saline before being sealed with bone wax. Care was taken to minimize bleeding and prevent extravasation of blood from the dura mater into the surgical field. A “C” or “T” shape incision was made on the dura mater, which was then flipped on the basal side and pulled open for fixation. Afterwards, endoscopic or microscopic surgery was performed.

**Figure 1:**
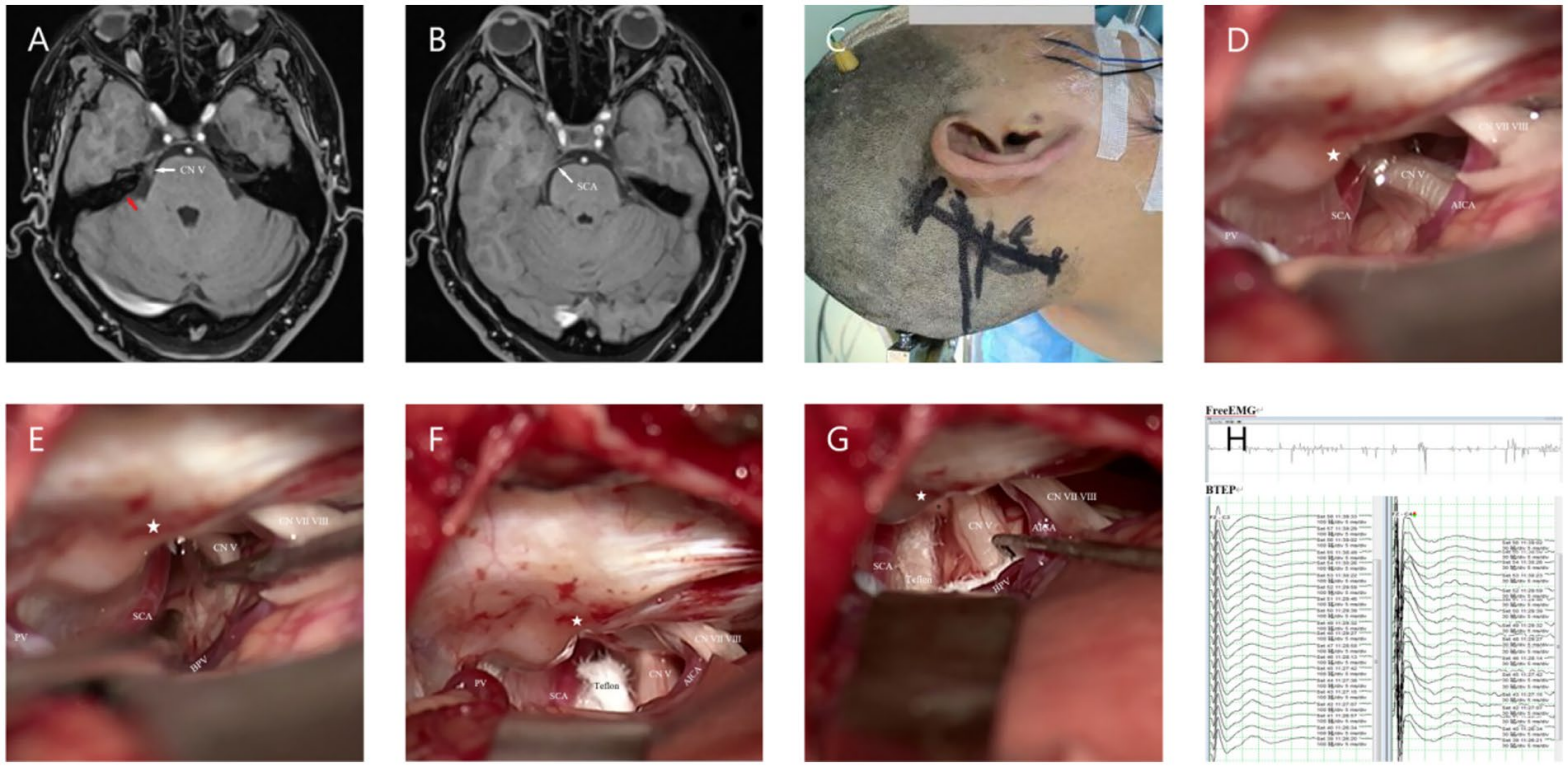
**(A)** Preoperative MRA (3D TOF-FS-SPGR MRA) reveals a relatively large PPT, indicated by the red arrow. **(B)** Combined with panel **(A)**, it can be seen that the SCA passes through from the ventral side above the Meckel’s cave (MC). **(C)** Longitudinal incision behind the right ear. **(D)** Under the microscope, it can be seen that the PPT (★) partially blocks the field of view, making it impossible to observe all nerves and vascular contacts. **(E)** Gently moving the nerve reveals the compression point located deep, posterior to the MC, which cannot be observed because of the PPT (★). **(F)** View after adjusting the operating table and microscope angle to observe the compression point. **(G)** Separating all possible offending vessels, * the first piece of Teflon cotton is filled, and the BPV is finally separated from the nerve. **(H)** Neuroelectrophysiological monitoring reveals an increase in the amplitude of the BTEP wave (not significant). CN V indicates trigeminal nerve (TN); CN VI VII, Facial and auditory nerve.

*The Microscopy (Zeiss T800, America) group (M-MVD)*: Cerebrospinal fluid (CSF) was slowly and fully released from the cerebellopontine angle (CPA) pool under microscopic guidance before gradually exploring the CPA area. After a satisfactory decrease in cerebral pressure, the arachnoid membrane was gradually separated using microscissors, exposing and freeing the facial-acoustic nerve and petrosal vein (PV) as much as possible to reduce nerve damage or bleeding caused by tension. The deep TN was then located before carefully identifying offending vessels from various angles to eliminate the possibility of omissions. Finally, Teflon pledgets were used for filling and separation, while changes in BTEP were observed. If the amplitude was not satisfactory, the Teflon pledget was adjusted or increased for further exploration. After a satisfactory increase in amplitude was observed, the surgery was concluded and the skull was gradually closed (Figure 1).

**Figure 2:**
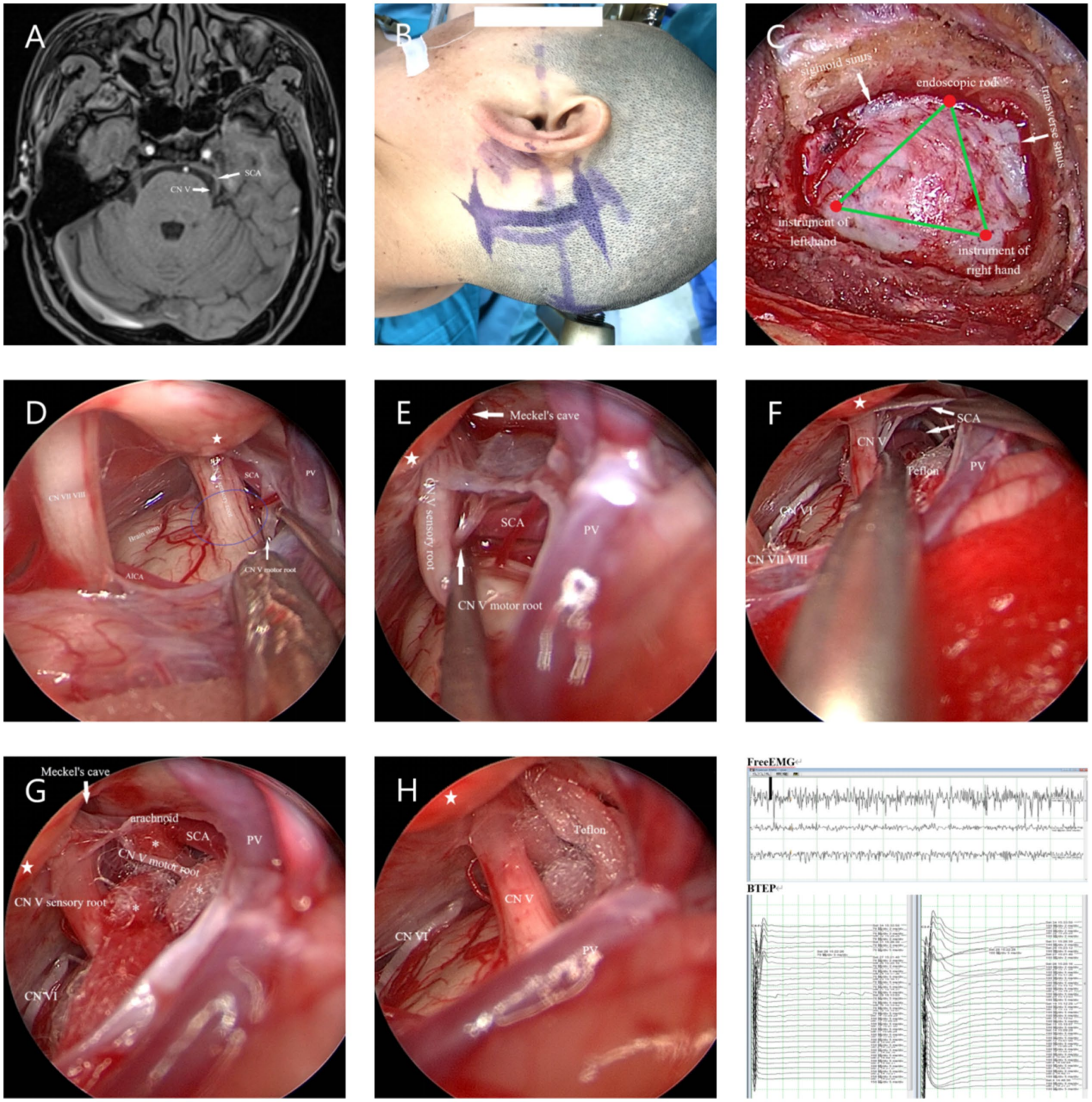
**(A)** Preoperative MRA indicated that the SCA passes through and compresses the TN on the ventral side above the position of the TN entering the MC. **(B)** Longitudinal incision behind the left ear. **(C)** The endoscope rod held by the assistant and the surgical instruments held by the primary surgeon create a triangular structure. **(D)** The SCA is seen compressing the TN from above, while the PPT (★) blocks further observation. The blue circle represents REZ of the TN. **(E)** The endoscope penetrates further and views the site where the nerve crosses the PPT (★) from above. Gently pulling open the TN reveals that the compression point is located on the ventral side behind the MC. **(F)** Further separation of the arachnoid membrane and using the Teflon pledget to open it further reveals that the SCA is shaped like a loop and compresses the TN. **(G)** From a higher perspective to assess the extent of decompression. **(H)** Post decompression. **(I)** Neuroelectrophysiological monitoring indicates a significant increase in BTEP waveform. CN VI, Abducens nerve.

*The Endoscopy (Karl Storz, Tuttlingen, Germany) Group (E-MVD):* First, a small amount of CSF was release from the CPA pool under direct vision. After a suitable amount of space was created, further operation was performed under endoscopy. The endoscope rod and the surgical instruments held by the primary surgeon create a triangular structure, with a 30° endoscope rod was hold by assistant positioned at the triangle’s vertex, and the surgical instruments of the primary surgeon’s hands located at the other two points (Figure 2C). After further releasing CSF, the entire TN is visualized from various angles using the 30° endoscope, allowing for careful identification of the offending vessels. The subsequent surgical steps are similar to those performed under a microscope (Figure 2).

*Neuroelectrophysiological monitoring and recording time*: BTEP changes were monitored throughout the surgery. BTEP changes were recorded before opening the dura mater, after sufficient release of CSF, before filling with Teflon pledgets, and after adequate neurovascular decompression.

### Outcome measures

#### Preoperative general information

The participants data, including gender, age, affected side, nerve affected, and preoperative MRA results, were compared between the two groups. Preoperative MRA results were jointly read by a team of five imaging and neurosurgery experts, and the most likely vessel was considered the offending vessel on preoperative MRA.

#### Intraoperative situation

Intraoperative findings that were compared between both groups included the length of the incision, diameter of the bone flap, surgical time, blood loss, consistency rate of preoperative-MRA result and intraoperative-offending vessel (CR-PMRA-IOV), whether the TN was fully exposed, and the rate of one-time decompression (ROD). The operation time was regarded as the time from the commencement of CSF release to the completion of neurovascular decompression during surgery. Blood loss was the sum of the blood volume in the drainage bag, the blood volume in the suction device, and the weight difference of the gauze after removing the volume of saline used to rinse the operative site. Regarding CR-PMRA-IOV, it was difficult to observe the PV using preoperative MRA; therefore, a mismatch caused by the PV and its branches during surgery was not considered a mismatch. The TN was fully exposed means that the entire TN structure from the brainstem to its entrance into the Meckel’s cave (MC) could be fully exposed during surgery, and we assessed it whether or not (Figures 1D,E, 2D–F). One-time decompression refers to the practice of not increasing or adjusting Teflon pledget after the initial electrophysiological monitoring, once sufficient filling has been completed. ROD assessed the probability of One-time decompression.

#### Postoperative evaluation

After surgery, a dedicated researcher evaluated the efficacy of the procedure at 1 week, 3 months, 1 year, 2 years, and at the last follow-up (using the BNI scores), and recorded the incidence of complications, recurrence rate at 2 years and the last follow-up, mortality rate, and length of hospital stay postoperatively. BNI scores of I and II were regarded as significant relief, a BNI score of III was regarded as relief, while BNI scores of IV and V were regarded as ineffective.

### Statistical methods

All data are presented as means ± standard deviation. Statistical analyses were performed using SPSS version 26.0 (IBM Corp., Armonk, NY, United States). The count data were analyzed using a t-test, while measurement data were analyzed using a Chi-square test. *p* values < 0.05 were considered statistically significant.

## Results

### Preoperative general information and results

There were no significant differences (*p* > 0.05) in gender, age, affected side and nerve, and preoperative MRA determination of the offending vessels between the two groups of patients (Table 1).

**Table 1:** Comparison of the general data between the two groups (*x–*±*s*, *n*, %).

Group		Total	Endoscopic group	Microscopic group	t/χ^2^ value	*p* value
Number of cases		131	53	78	-	-
Gender	Male	43 (32.82)	15 (28.30)	28 (35.90)	0.826	0.364
	Female	88 (67.18)	38 (71.70)	50 (64.10)		
Age	Years	56.49 ± 7.64	57.04 ± 8.63	56.12 ± 6.91	0.676	0.501
Disease duration	Months	46.48 ± 35.37	44.19 ± 32.15	48.04 ± 37.52	1.129	0.261
Side	Left	49 (37.40)	17 (32.08)	32 (41.03)	1.080	0.299
	Right	82 (62.60)	36 (67.92)	46 (58.97)		
Nerve affected	V1	8 (6.11)	4 (7.55)	4 (5.13)	1.895	0.997
	V2	51 (38.93)	22 (41.51)	29 (37.18)		
	V3	36 (27.48)	15 (28.30)	21 (26.92)		
	V1, V2	12 (9.16)	3 (5.66)	9 (11.54)		
	V2, V3	22 (16.79)	8 (15.09)	14 (17.95)		
	V1, V2, V3	2 (1.53)	1 (1.89)	1 (1.28)		
Offending vessel of MRA	SCA	81 (61.83)	28 (52.83)	53 (67.95)	3.739	0.988
	AICA	14 (10.69)	7 (13.21)	7 (8.97)		
	PICA	3 (2.29)	2 (3.77)	1 (1.28)		
	PV/BPV	0 (0)	0 (0)	0 (0)		
	VA	7 (5.34)	4 (7.54)	3 (3.85)		
	BA	2 (1.53)	1 (1.89)	1 (1.28)		
	MVC	2 (1.53)	1 (1.89)	1 (1.28)		
	None	22 (16.79)	10 (18.87)	12 (15.39)		

SCA, Superior cerebellar artery; AICA, Anterior inferior cerebellar artery; PICA, Posterior inferior cerebellar artery; PV, Petrosal vein; BPV, Branch of Petrosal vein; VA, Vertebral artery; BA, Basilar artery; MVC, Multiple vascular compression; None, No vascular compression.

### Intraoperative findings

There were differences in the incision lengths and bone flap diameters between the two groups (*p* < 0.05); however, there was no significant difference in the total surgical time and blood loss between the two groups (*p* > 0.05) (Table 2).

**Table 2:** Comparison of partially intraoperative findings between the two groups (*x–*±*s*).

Group		Endoscopic group	Microscopic group	t/χ^2^ value	*p* value
Number of cases		53	78		
Length of incision	cm	3.97 ± 0.38	5.10 ± 0.36	17.241	0.000
Diameter of bone flap	cm	2.51 ± 0.22	3.09 ± 0.18	16.530	0.000
Total surgical time	min	27.08 ± 9.05	28.31 ± 8.52	0.791	0.431
Blood loss	mL	47.74 ± 16.37	50.64 ± 19.97	0.939	0.349

After dividing the patients in both groups into the neurovascular compression (NVC) group and the no neurovascular compression (NNVC) group based on preoperative MRA results, no significant differences were observed in surgical time between patients in the NVC and NNVC groups who underwent E-MVD and between the NVC group of patients that underwent either endoscopic or M-MVD (*p* > 0.05). However, there were differences in surgical time between the NVC group and NNVC group among patients that underwent M-MVD and the NNVC group of patients that underwent endoscopic or M-MVD (*p* < 0.05) (Table 3). The results indicate that for patients without significant neurovascular compression on preoperative MRA examination, endoscopic treatment was relatively faster; this was due to faster identification and determination of the offending vessel during E-MVD.

**Table 3:** Comparison of surgical time of NVC and NNVC between the two groups (*x–*±*s*, *n*).

Group		Total	Endoscopic group	Microscopic group	t value	*p* value
Number of cases		131	53	78		
Surgical time (min)	NVC	43 + 66	26.53 ± 9.33	26.18 ± 6.04	0.238	0.812
	NNVC	10 + 12	29.4 ± 7.75	40 ± 10.78	2.596	0.017
t value			0.901	6.355		
*p* value			0.372	0.000		

NVC, neurovascular compression; NNVC, no neurovascular compression.

There was no statistically significant difference in the intraoperative offending vessels between the two groups (*p* > 0.05); however, there was a difference in the CR-PMRA-IOV between the two groups (*p* < 0.05), indirectly indicating that endoscopy was more effective than microscopy in detecting offending vessels. There was a significant difference in the ability to fully expose the TN and the ROD between the two groups (*p* < 0.05). Compared to M-MVD, E-MVD was more effective in fully exposing the TN and was associated with fewer adjustments or increases of the Teflon pledget after neuroelectrophysiological monitoring verification and decompression (Table 4).

**Table 4:** Comparison of other partially intraoperative findings between the two groups (*n*, %).

Group		Total	Endoscopic group	Microscopic group	t/χ^2^ value	*p* value
Number of cases		131	53	78	-	-
Offending vessel	SCA	88 (67.18)	34 (64.15)	54 (69.23)	3.324	0.998
	AICA	8 (6.11)	4 (7.55)	4 (5.13)		
	PICA	1 (0.76)	0 (0)	1 (1.28)		
	PV/BPV	8 (6.11)	4 (7.55)	4 (5.13)		
	VA	7 (5.34)	4 (7.55)	3 (3.85)		
	BA	1 (0.76)	0 (0)	1 (1.28)		
	MVC	14 (10.69)	6 (11.32)	8 (10.25)		
	None	4 (3.05)	1 (1.88)	3 (3.85)		
CR-PMRA-IOV	Yes	98 (74.81)	34 (64.15)	64 (82.05)	5.366	0.021
	No	33 (25.19)	19 (35.85)	14 (17.95)		
CE-TN	Yes	87 (66.41)	51 (96.23)	36 (46.15)	33.261	0.000
	No	44 (33.59)	2 (3.77)	42 (53.85)		
ROD	Yes	109 (83.21)	49 (92.45)	60 (76.92)	4.392	0.036
	No	22 (16.79)	4 (7.55)	18 (23.08)		

CR-PMRA-IOV, the consistency rate of Preoperative-MRA result and Intraoperative-offending vessel; CE-TN, completely expose the TN; ROD, the rate of one-time decompression.

### Postoperative results

As shown in Table 5, the rate of significant relief after E-MVD was 92.45%, while that after M-MVD was 87.18% at 1 week postoperatively. Furthermore, the significant relief rate after E-MVD was 84.91%, while that after M-MVD was 79.49% at 2 years postoperatively (Table 5). There was no significant difference in postoperative efficacy between the two groups (*p* > 0.05) (Table 6).

**Table 5:** Comparison of clinical treatment efficacy between the two groups (*n*, %).

Group		Total	Endoscopic group	Microscopic group
Number of cases		131	53	78
Preoperative BNI scores	IV	8 (6.11)	3 (5.66)	5 (6.41)
	V	123 (93.89)	50 (94.34)	73 (93.59)
1 week postoperative	I	101 (77.10)	44 (83.02)	57 (73.08)
	II	16 (12.21)	5 (9.44)	11 (14.10)
	III	8 (6.11)	2 (3.77)	6 (7.69)
	IV	6 (4.58)	2 (3.77)	4 (5.13)
	V	0 (0)	0 (0)	0 (0)
3 month postoperative	I	108 (82.44)	46 (86.79)	62 (79.49)
	II	12 (9.16)	4 (7.55)	8 (10.25)
	III	5 (3.82)	1 (1.89)	4 (5.13)
	IV	6 (4.58)	2 (3.77)	4 (5.13)
	V	0 (0)	0 (0)	0 (0)
1 year postoperative	I	103 (78.63)	44 (83.02)	59 (75.64)
	II	11 (8.40)	4 (7.55)	7 (8.97)
	III	4 (3.05)	2 (3.77)	2 (2.57)
	IV	9 (6.87)	2 (3.77)	7 (8.97)
	V	4 (3.05)	1 (1.89)	3 (3.85)
2 year postoperative	I	98 (74.81)	43 (81.14)	55 (70.52)
	II	9 (6.87)	2 (3.77)	7 (8.97)
	III	6 (4.58)	2 (3.77)	4 (5.13)
	IV	9 (6.87)	4 (7.55)	5 (6.41)
	V	9 (6.87)	2 (3.77)	7 (8.97)
Last follow-up	I	97 (74.05)	43 (81.13)	54 (69.23)
	II	8 (6.11)	1 (1.89)	7 (8.97)
	III	4 (3.05)	2 (3.77)	2 (2.57)
	IV	7 (5.34)	3 (5.66)	4 (5.13)
	V	15 (11.45)	4 (7.55)	11 (14.10)

**Table 6:** Comparison of clinical treatment efficacy between the two groups (*x–*±*s*).

Group	Endoscopic group	Microscopic group	t/χ^2^ value	*p* value
BNI scores				
Preoperative	4.94 ± 0.23	4.93 ± 0.24	0.238	0.812
1 week	1.28 ± 0.72	1.45 ± 0.85	1.194	0.235
3 month	1.23 ± 0.67	1.36 ± 0.81	0.965	0.336
1 year	1.34 ± 0.88	1.56 ± 1.15	1.178	0.241
2 year	1.49 ± 1.12	1.74 ± 1.33	1.124	0.263
Last follow-up	1.57 ± 1.26	1.86 ± 1.48	1.167	0.245

There were no deaths or serious complications in both groups of patients postoperatively. There were also no differences in the incidence of complications and length of postoperative hospital stay (*p* > 0.05). The 2-year and last follow-up recurrence rates in the endoscopic group were lower than those in the microscopic group (5.88% vs. 12.16 and 7.84% vs. 16.22%, respectively), but the difference was not statistically significant (*p* > 0.05). A longer follow-up time may lead to different results (Table 7).

**Table 7:** Comparison of postoperative complication and recovery between the two groups (*x–*±*s*, *n*, %).

Group		Total	Endoscopic group	Microscopic group	t/χ^2^ value	*p* value
Number of cases		131	53	78	-	-
Complication	Facial numbness	8 (6.11)	3 (5.66)	5 (6.41)	0.038	0.845
	Facial paralysis	2 (1.53)	1 (1.89)	1 (1.28)	0.201	0.654
	Orofacial herpes	3 (2.29)	2 (3.77)	1 (1.28)	0.116	0.733
	Hypoacusis	4 (3.05)	1 (1.89)	3 (3.85)	0.015	0.903
	Diplopia	2 (1.53)	1 (1.89)	1 (1.28)	0.201	0.654
	Headache/dizziness/nausea and vomiting	10 (7.63)	2 (3.77)	8 (10.26)	1.074	0.300
	Intracranial infection	3 (2.29)	2 (3.77)	1 (1.28)	0.116	0.733
	Subcutaneous hydrops	2 (1.53)	0 (0)	2 (2.56)	1.380	0.502
	Total cases	25 (19.08)	9 (16.98)	16 (20.51)	0.255	0.614
2-year recurrence rate	Yes	12 (10.62)	3 (5.88)	9 (12.16)	0.744	0.388
	No	113 (89.38)	48 (94.12)	65 (87.84)		
Last follow-up recurrence rate	Yes	16 (12.8)	4 (7.84)	12 (16.22)	1.220	0.269
	No	109 (87.2)	47 (92.16)	62 (83.78)		
Postoperative hospital stay	(days)	6.64 ± 1.83	6.74 ± 2.17	6.58 ± 1.58	0.488	0.626

## Discussion

Although vascular contact in the root entry zone (REZ) is widely regarded as the main cause of PTN, an increasing number of studies have shown that vascular compression at any point in the intracranial segment of the TN can lead to PTN (6, 9). Therefore, maximizing exposure of the intracranial segment of the TN which is located deep in CPA, is the key to finding the site of vascular compression and achieving surgical success (6, 10, 11). The field of view of the microscope is limited to the various angles observable outside the dura mater. However, endoscopy can bring the field of view into the CPA cavity. A better visualization and a panoramic view of the CPA area can help surgeons see hidden sections after slightly pulling the brain tissue that are inaccessible to a microscope (6, 10–13). Tang et al. (14) found that the field of view provided by a 30° endoscope was almost twice that provided by a microscope, which means that in order to achieve or approach the field of view of the endoscope while using a microscope, a larger bone flap needs to be created or the cerebellar tissue needs to be stretched to a greater extent (6, 10, 11). We found that the incision length and bone flap size required for E-MVD were smaller than those required for M-MVD (*p* < 0.05), which is consistent with previous report (6, 10, 15, 16). Halpern et al. (16) used a bone flap with a diameter of approximately 1 cm and successfully completed 38 E-MVD surgeries with satisfactory results. However, according to Charalampaki et al. (10), the keyhole approach does not mean that the skull opening is a hole, but rather the correct individualized choice to enter key areas for operation with minimal trauma and maximum efficiency. Endoscopy also requires a certain amount of operating space; therefore, we choose a bone flap with a diameter of approximately 2 cm according to individual circumstances, which is beneficial for reducing mutual interference of instruments intraoperatively while also reducing surgical time, injury, and postoperative complications.

Even in patients with a significant petrosal protuberance tubercle (PPT), endoscopy can still visualize the position of the distal TN up to the MC, which is difficult to achieve under the microscope’s field of view (6, 10, 17). Our research also supported this viewpoint. When the PPT obstructs the surgical field, the microscope can only be used after rotating the operating table and adjusting the angle of view; moreover, this may not always be feasible or effective (Figures 1D–G). However, in a similar scenario, an endoscope can easily elevate the raised bone ridge and explore the MC area of the TN. Similarly, the lens can be rotated to easily explore the surgical site and operate with less difficulty (Figures 2D–G). Therefore, compared to microscopes, endoscopes are more efficient in identifying hidden vascular conflicts, such as the ventral part of the REZ or MC (6, 10). Chen et al. (18) found that approximately 14.74% of the offending vessels in 167 patients with PTN were missed during M-MVD and only discovered during endoscopy. Furthermore, Luzzi et al. (19) reported that out of 43 cases of synchronous endoscopic and microscopic surgeries, nine cases (21%) that involved compression of one or more vessels on the ventral side of the nerve were missed during M-MVD but detected using endoscopy, while six cases (14%) could not be observed and operated on using the microscope but were operated using endoscopy. According to Charalampaki et al. (10), endoscopy has also been reported to detect almost 100% of offending vessels even under multi-vessel compression. These studies suggest that using endoscopy increases the likelihood of identifying offending vessels.

**Figure 3:**
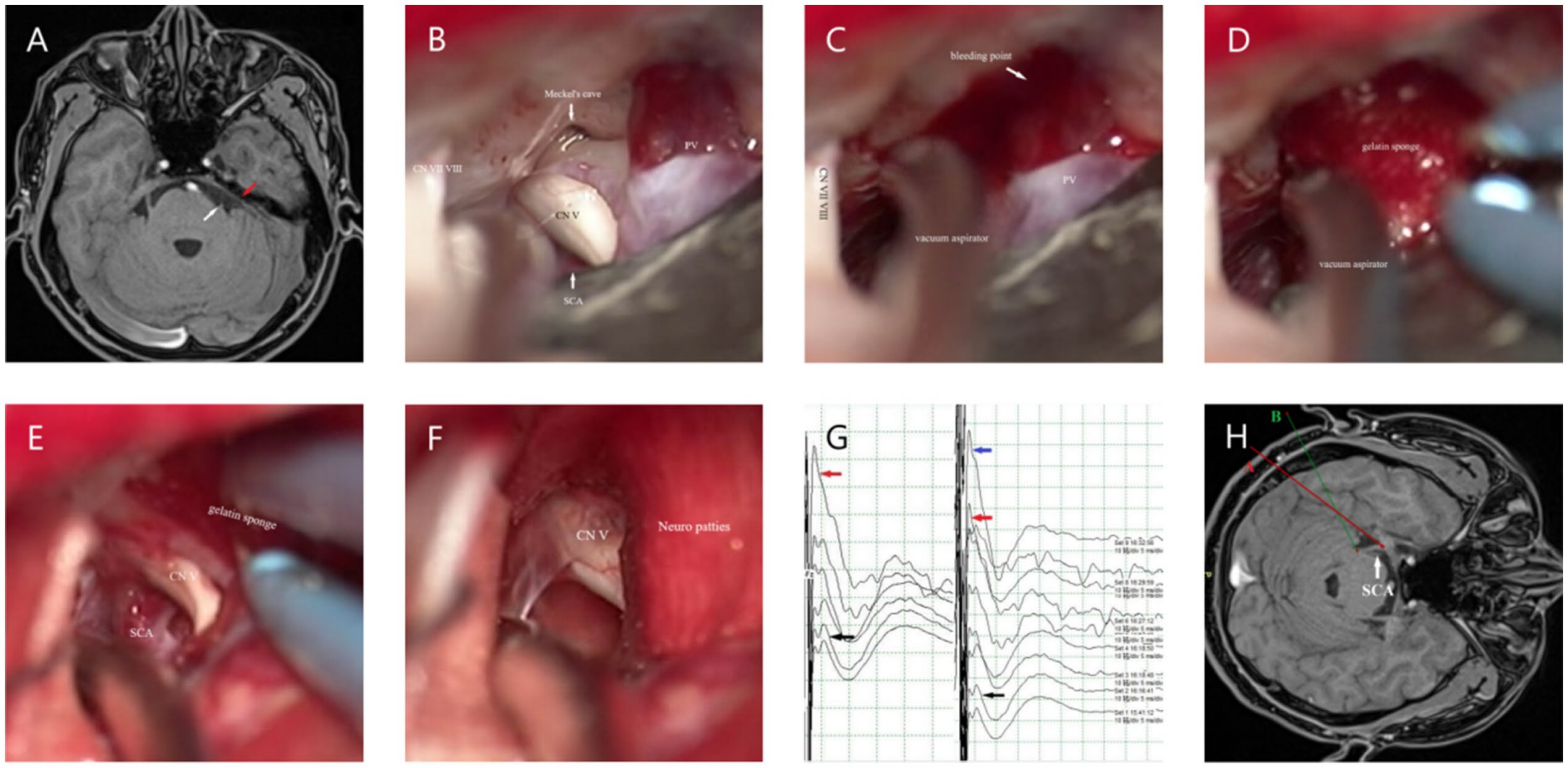
**(A)** The white arrow represents the starting point from the origin of the TN in the brainstem. MRA shows no obvious PPT (the red arrow). **(B)** The MC can be clearly seen under the microscope if the PPT is not obvious. **(C)** When separating the surrounding arachnoid membrane, the PV is pulled and bleeding occurs; the vacuum aspirator is used to attract it on the side and maintain a clear field of view. **(D)** The gelatin sponge is used to stop the bleeding quickly. **(E,F)** After using gelatin sponge and neuro patties, bleeding from the PV was arrested. **(G)** The black arrow shows the BTEP waveform before opening the dura mater, and the red arrow shows the waveform after CSF release, which is significantly higher than before. The blue arrow indicates that the waveform further increases after sufficient decompression. **(H)** When checking the position of the MC, it can be seen from the angle of the A line, while checking the REZ, it can be seen from the angle of the B line.

In our study, there was a difference in the CR-PMRA-IOV between the two groups (*p* < 0.05). The endoscopic group had a lower overall accuracy, but this may suggest that endoscopy is better at correctly identifying offending vessels due to its ability to detect subtle abnormalities that might be overlooked with microscopy. Although attempts were made to exclude personal factors, it was not an objective indicator and had limited reference significance. Compared to microscopes, endoscopes are more capable of fully exposing the TN (*p* < 0.05). In our cases, endoscopes were capable of observing the entire intracranial segment of the TN in almost all patients, and only a few patients were not forcibly separated for observation due to challenges such as occlusion of the PV. However, microscopes were used to fully expose the TN in only 46.15% of patients. This was mainly due to occlusion of the PPT or the short length of the PV, which complicated separation. Additionally, in patients with high cerebral pressure the surgical cavity was narrower; this challenge was more common in younger patients. When the PPT is not prominent or the petrous bone is very flat, the MC area can also be observed using a microscope (Figures 3A,B); therefore, it is important to carefully analyze the imaging characteristics of the patient preoperatively. With higher ROD in the endoscopic group (*p* < 0.05), which represented less common to adjust the Teflon pledget after filling according to neuroelectrophysiological results, indicated that decompression under endoscopic visualization was more direct and reduced the number of nerve disturbances. We also found that there was no significant difference in the surgical time between the two groups which is consistent with the report of previous studies (16). However, when the patients were divided into the NVC and NNVC groups based on preoperative MRA results, we found that using an endoscope led to a shorter surgical time in the NNVC group compared to using a microscope (*p* < 0.05). This is mainly because when searching for the offending vessel, endoscopy can closely and multi-angular observe the entire structure of the TN, leading to faster determination of the offending vessel, more efficient decompression, and ultimately a shorter surgical time. However, microscopes can only search for possible offending vessels from various angles along the TN to a limited extent, often requiring repeated verifications using neuroelectrophysiological reactions.

Although the benefits of endoscopy in surgery are obvious due to the advantages of imaging systems, there is still much controversy over the postoperative efficacy and probability of complications. Pak et al. reported on 25 patients PTN who underwent E-MVD, all of whom were pain-free immediately after surgery. However, two patients experienced hearing loss after surgery, while one patient had transient facial paralysis. During follow-up, 22 (88%) of the patients remained pain-free (20). Charalampaki et al. (10) reported on 35 patients who underwent E-MVD, and during a 6-month follow-up period, 34 cases (98%) remained completely pain-free, with only one patient experiencing CSF leakage. Overall, these reports have demonstrated the effectiveness and safety of E-MVD. The findings of Kabil et al. (13) suggest that compared to M-MVD, E-MVD was associated with higher pain relief rates, lower incidence of complications, and better outcomes, as well as Li’s report (12). Zagsoog et al. (21) conducted a comparative meta-analysis, and showed that M-MVD and E-MVD have similar clinical outcomes in terms of pain relief. E-MVD was also shown to be associated lower recurrence rates, lower incidence rates of complications, as well as other advantages related to its shorter surgical time and more minimally invasive nature.

However, other reports have found no significant differences between endoscopy and microscopy in terms of pain relief rate, recurrence rate, and incidence of complications (16, 22, 23). Our research findings appear to support this viewpoint, as we found no significant differences in postoperative follow-up remission rate, BNI scores, the incidence of postoperative complication, recurrence rate, mortality rate, and postoperative hospital stay between the two groups (*p* > 0.05). However, in our study, the recurrence rate at 2 years postoperatively and the last follow-up was lower in the endoscopic group compared to the microscopic group, but the difference was not significant (*p* > 0.05). Furthermore, a longer follow-up duration may lead to different results. Since endoscopy has obvious imaging advantages, why then do such results occur? We believe there may be two reasons. Firstly, all patients in our study underwent neuroelectrophysiological monitoring, and the Teflon pledget was adjusted in a timely manner interoperatively based on the results of neuroelectrophysiological monitoring to achieve satisfactory results. Secondly, the compression site was located in the MC area, and the PPT was significant, or the ventral area of REZ could not be observed using the microscope in only a few cases.

### The use of intraoperative neuroelectrophysiological monitoring

Research has found that the BTEP in patients with PTN is characterized by disappearance of the evoked potential, poor differentiation, prolonged latency, and decreased amplitude, with a sensitivity of up to 100%. After decompression, waveform recovery or an increase in the observed amplitude occurs, and this is a sensitive indicator for monitoring the conduction function of the TN, which has a direct predictive effect on the postoperative efficacy of MVD (24–26). Therefore, in our study, the degree of change in BTEP amplitude was used as a criterion for determining whether adjustments or the addition of Teflon pledgets were needed intraoperatively. In our experience, an increase in amplitude of more than 50% indicates good outcomes, while an increase in amplitude of less than 50% may indicate poor results and the need to adjust the Teflon pledget. However, this is not absolute. Interestingly, we found that in some patients, after the release of CSF intraoperatively, there was an improvement in the amplitude of the BTEP. This may be due to an increase in the surrounding space and gaps between blood vessels and nerves after the release of CSF, which reduces the impact of arterial pulsation on the TN (Figure 3G).

### Our center’s experience in endoscopic and microscopic surgery

#### Microscopic surgery

First, we attempt to make a slightly larger bone flap, especially in cases where the preoperative offending vessels are unclear or multiple offending vessels are present; Second, during the operation, adjustments are made to the operating table and various angles of the microscope (Figure 3H) to adequately expose the entire TN. The PPT can be removed when it obstructs the view (27); however, this may increase the risk of postoperative CSF leakage and infection. Third, the release of CSF should be sufficient, and the arachnoid space around the facial-acoustic nerve and PV should be fully freed as much as possible to reduce the tension of subsequent decompression operations. Otherwise, once the PV tears and bleeds, subsequent operations will become more difficult; however, in some cases, PV injury may be unavoidable (Figures 3C–F). First, a vacuum aspirator is used to quickly and lightly aspirate blood from the operative field. A vacuum aspirator should not be used to block the bleeding site as is done in the treatment of ruptured aneurysms, because the venous wall is thin, and strong suction can easily enlarge the bleeding site and cause more aggressive bleeding. Then, a gelatin sponge and neuro patties are quickly applied to the bleeding site to compress the bleeder to achieve hemostasis. If the bleeding site is small, a low-power electrocoagulation sponge can be used to achieve hemostasis, similar to an ‘artificial thrombosis.’ If bleeding is difficult arrest, the petrosal vein can be sacrificed; however, it is important to be aware of the possibility of postoperative cerebellar swelling. If the cerebral pressure is high, prophylactic resection of the lateral cerebellum can be considered intraoperatively. Teflon pledgets are hard; therefore, if PV is the responsible vessel, during decompression we often use small pieces of gelatin sponge to protect the responsible vein before using Teflon pledgets for decompression. After decompression, the gelatin sponge is usually left *in situ*.

#### Endoscopic surgery

In the early days of E-MVD at our center, the use of an auxiliary arm was necessary. However, over our years of endoscopic application, we have found that the auxiliary arm may not synchronize effectively with the surgeon. The continuous adjustment often increased the operative time. Therefore, if there is an experienced assistant, it is recommended to use an assistant to hold the endoscope. A skilled assistant can significantly shorten the operation time, and the surgeon can operate more comfortably and naturally. Although a smaller bone flap appeared more minimally invasive, we encountered more intraoperative difficulties, and the surgical instruments were prone to interference, which may increase the risk of internal nerve and vascular damage. We recommend a slightly larger bone flap which can achieve a balance between minimally invasive access and safety. Additionally, it is recommended to use a 30° lens, which can balance both the front and partial side views while allowing unrestricted operation. After decompression, the 30° lens can evaluate the operative site from multiple angles and assess whether the Teflon pledget is correctly placed and whether the decompression is sufficient. We believe that under endoscopic guidance, extensive separation of the arachnoid membrane is not always beneficial, especially when the distal end of the TN is compressed at the MC, which is relatively spacious. Retaining a portion of the arachnoid membrane can be useful when fixing the Teflon pledget and preventing it from falling off during postoperative CSF fluctuations (Figures 2F,G). As reported by Halpern et al. (16), it is not recommended to transition directly from microscopy to endoscopy. Our center advanced from microscopic surgery to endoscopic-assisted microscopy surgery, and then to fully endoscopic surgery. Luzzi et al. (19) reported that combining the use of a microscope and an endoscope for observation and manipulation can reduce their respective shortcomings; however, such an arrangement is also associated with its own drawbacks, including the need for more instruments, a cumbersome operation, and higher requirements for surgeons.

From our center’s previous experience, we found that repeated entry and exit of instruments and blood smudges on the lens can increase surgical time. Therefore, especially in endoscopic procedures, meticulous hemostasis is essential prior to exploration and operation. The requirements for handling intraoperative vascular bleeding are fast and precise, which can greatly challenge a surgeon’s proficiency with endoscopic tools. Therefore, endoscopic surgery is more difficult. All attempts should be made to avoid large movements or instrument contact with the lens intraoperatively. It is our opinion that the main learning difficulty of endoscopy is the 3D-2D transition. Endoscopy can only observe what is in front of the lens and has poor depth perception. When the instrument passes through the posterior channel, it can easily damage surrounding tissues. We recommend that every time an instrument is to be replaced, the lens should be retracted to a relatively safe area before the instrument is inserted. This requires a relatively long adaptation and practice. Once the learning curve is overcome, endoscopy can achieve operational accuracy similar to that of a microscope. Additionally, among patients who are obese and young, the cerebellum is more prone to swelling after opening the dura mater, which affects further endoscopic procedures. To address this issue, we will take measures such as raising the head, administering mannitol and furosemide. However, the more critical solution is to fully drain CSF, as endoscopic use in very narrow spaces can easily damage brain tissue. In this series of cases, patients in the endoscopic group obtained sufficient exposure space through this method, and no patients required additional use of retractors and lumbar drainage. Finally, thermal damage caused by endoscopy cannot be ignored. Therefore, we suggest that when searching for the offending vessels, the surgeon should try to get as close as possible to avoid missing them. However, during the decompression process, the surgeon needs to limit contact with blood vessels and neural tissues to reduce the risk of thermal and mechanical damage caused by blind spots in the field of view.

#### Individualized treatment

An increasing number of studies have found that the postoperative efficacy and incidence of complications of E-MVD and M-MVD are similar. Currently, there is no evidence indicating that endoscopy can replace microscopy for MVD surgery. Our study’s findings also appear to arrive at the same conclusion. Therefore, regarding patient selection, it is our opinion that more attention should be paid to individualized treatment plans for patients. Preoperatively, we should conduct a comprehensive evaluation based on the patient’s age, preoperative CT, MRA imaging characteristics, and other factors. In our center, we believe that the following points are more suitable for endoscopic surgery: 1. Preoperative MRA does not show significant neurovascular compression, or preoperative images show very small arteries passing through the ventral area of REZ or the entrance of the MC; 2. Preoperative imaging suggests the presence of a large PPT, which may affect microscopic observation intraoperatively; and 3. In relatively young patients with fuller brain tissue, it may be difficult to effectively achieve an adequate surgical space intraoperatively. In these cases, the wide and good field of view of endoscopy can facilitate the detection of the possible offending vessels and reduce omissions caused by limitation of the viewing angle. However, surgeons should select surgical methods that we are more confident in.

### Limitation

This study has some limitations: (1) this study’s retrospective design lacks the random allocation achievable in prospective studies. Patient assignment to the endoscopic or microscopic group primarily depended on individual preferences, and the surgeon did not perform randomized allocation or interventions. These factors may have introduced selection bias, potentially affecting the reliability and generalizability of our results. (2) Another limitation of this study is its relatively short follow-up duration. In the future, we hope to conduct further studies with long-term follow-up periods of 5 or 10 years. (3) Finally, this study is a single-center investigation with a limited sample size, which inherently presents certain limitations. We look forward to the results of future research conducted through multi-center collaborations.

## Conclusion

E-MVD and M-MVD are both safe and effective for PTN and have comparable outcomes. Endoscopy enhances visualization and identification of offending vessels; moreover, compared with microscopy, endoscopy is more minimally invasive and suitable for detecting and fully separating potentially hidden offending vessels. However, the technical difficulty of endoscopy is higher, and each center should choose personalized treatment plans based on the individual situation of patients and their respective technical advantages.

## Data Availability

The raw data supporting the conclusions of this article will be made available by the authors, without undue reservation.
